# The ICU environment paradigm as a catalyst for better delirium models

**DOI:** 10.1042/CS20260577

**Published:** 2026-05-14

**Authors:** Zyad J. Carr

**Affiliations:** Department of Anesthesiology, Division of Critical Care Medicine, Yale University School of Medicine, New Haven, Connecticut, U.S.A.

**Keywords:** Delirium, ICU delirium, ICU like environment, Intensive care, Translational Model

## Abstract

The present commentary argues that incorporating ICU-like environmental stressors into animal delirium models improves translational validity. By combining surgery or sepsis with light, noise, sleep disruption, and handling, the discussed paradigm better reflects human ICU delirium, supports mechanistic discovery, and highlights circadian disruption as a promising framework for future investigation.

Delirium is a common and devastating acute brain dysfunction in critically ill patients, tightly linked to increased morbidity, mortality, institutionalization, and long-term cognitive decline [1–3]. Yet despite decades of observational and interventional clinical work, its neurobiology remains elusive. A major obstacle has been the lack of robust, translationally relevant animal models. Their manuscript, ‘The ICU environment paradigm as a strategy to enhance the validity of delirium animal models,’ clearly advances the field by explicitly incorporating an ICU-like environment into established delirium-related paradigms and by carefully addressing face, construct, and predictive validity [4] (see Figure 1).

**Figure 1:**
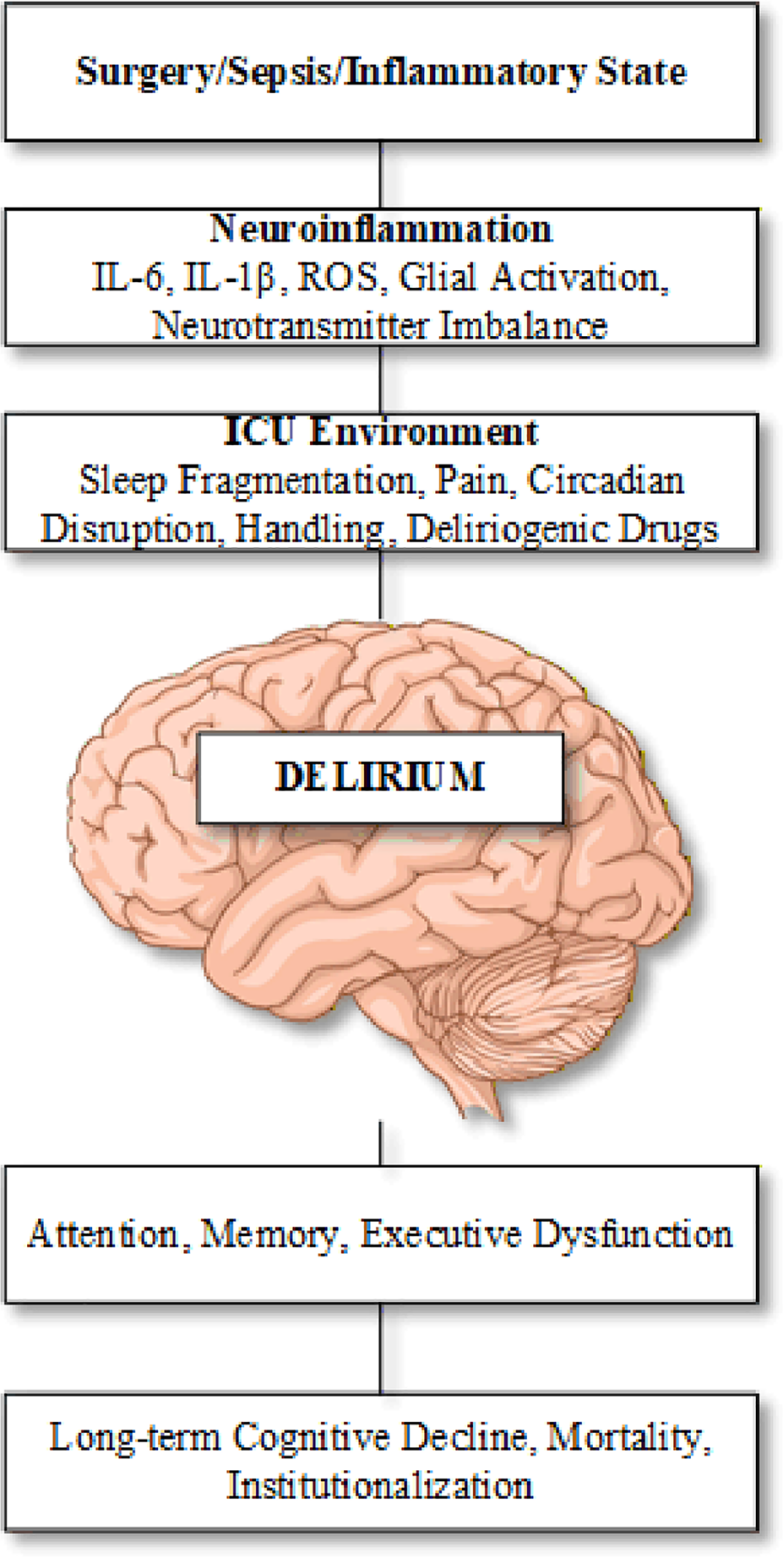
The ICU Delirium Triad. The ICU Delirium Triad. Three converging pathogenic domains, precipitating insult, neuroinflammation, and ICU environment, best characterize the complexity of ICU-related delirium. The sequelae and cost to society are serious: increased morbidity, mortality, and long-term institutionalization.

Historically, preclinical delirium research has leaned heavily on models based on major surgery, infection, or systemic inflammation [5,6]. These insults are clinically relevant, but they occur within a much more complex and detrimental environmental context in ICU patients: unrelenting light and noise, repeated physical disturbances, sedation, sleep fragmentation, and invasive monitoring, which prior models have inadequately captured [7]. Dominguini et al. explicitly recognize this gap, noting that current models have not, to date, earnestly taken into consideration the ICU environment, with most relying mainly on surgical insult, infection, or inflammatory insult to elicit a delirium-like state.

Their key innovation is to integrate an ICU-like environmental paradigm into two widely used models: sterile surgery with anesthesia (laparotomy) and CLP-induced sepsis. Importantly, they do so with explicit attention to multi-dimensional validity. Face validity was achieved using paradigms such as sterile laparotomy (surgery) and cecal ligation and puncture (sepsis) that reproduced acute impairments in attention, memory, and executive function akin to human delirium. Construct validity was ensured by exposing animals to systemic infection, anesthesia, surgical stress, and ICU-like environmental stressors, well-recognized etiologies for ICU delirium [8]. Predictive validity was demonstrated by showing that pharmacological agents attenuated behavioral, inflammatory, and neurochemical alterations. This explicit, structured approach to validity is entirely in line with recent recommendations for delirium models, including those articulated by Vasunilashorn et al. in the Network for Investigation of Delirium: Unifying Scientists (NIDUS) network review [9].

## How the ICU-like environment is modeled

The methods used to operationalize the ICU-like environment are worth highlighting, as they attempt to approximate the sensory and physical stressors common in critical care. Male Wistar rats first underwent either laparotomy (under isoflurane anesthesia) or CLP-induced sepsis. For the laparotomy model, the authors describe a standard laparotomy protocol. To this, they add an ICU-like paradigm in a subgroup consisting of propofol administration (50–100 mg/kg intraperitoneally) followed by 12 h of an ICU environment, consisting of exposure to a 33 W light, 90 dB sounds, and cage rattling via a horizontal shaker (120 rpm) for 1 min every 20 min. This ICU-like environment incorporates typical environmental stressors commonly encountered by patients in the ICU, such as constant illumination, persistent alarms and machinery sounds, and frequent physical disturbances, enhancing the translational relevance of the model to human ICU conditions [10]. Crucially, this ICU-like exposure is layered on top of clinically meaningful insults (surgery or sepsis), addressing a key criticism of earlier ICU-paradigm studies that did not apply comparable stressors to control groups. As they note, prior work ‘incorporated the ICU-like environment into the laparotomy model without applying any comparable stressor to the control animals,’ leaving open questions about construct and internal validity. The present design directly addresses this concern.

The behavioral characterization in the present study uses multiple tests within the first 36 h after insult in a time window relevant to acute delirium onset in human patients. The authors employ the open-field, Y-maze, and SmithKline Beecham, Harwell, Imperial College, Royal London Hospital phenotype tests, which together probe locomotion, attention/short-term memory, and a range of behavioral domains. They also estimate delirium incidence/severity using composite behavioral criteria, reflecting an effort to move beyond single-task readouts and toward a more syndrome-like definition. While there is ongoing debate about how best to operationalize delirium in animals, this multidomain approach is aligned with the field’s emphasis on complex, fluctuating disturbance in attention, arousal, and cognition rather than mere sickness behavior or sedation.

## Neuroinflammation, glial activation, and neurotransmitter imbalance

On the mechanistic side, Dominguini et al. concentrate on domains that have repeatedly emerged in human and animal delirium research: inflammation, glial responses, oxidative stress, and neurotransmitter imbalance [11–13]. They report elevations in IL-6 (hippocampus, cortex, and plasma in laparotomy; hippocampus in sepsis) and IL-1β (cortex in laparotomy), alongside aminergic/excitatory imbalance (dopamine, noradrenaline, and glutamate). The spatially specific cytokine elevations (hippocampus and cortex) are notable, as these regions are central to attention and memory; core domains affected in delirium. The concurrent aminergic and glutamatergic alterations further support the long-standing hypothesis that delirium arises from a dysregulated balance between cholinergic, aminergic, and glutamatergic systems [14]. In addition, they quantify microglial markers and reactive oxygen species. They show that the ICU-like environment worsens neuroinflammatory and oxidative profiles beyond surgery or sepsis alone and that interventions can modulate these changes.

These findings not only support construct validity (involving hypothesized delirium mechanisms) but also provide footholds for mechanistic dissection, e.g., differential roles of microglia, oxidative stress, and cholinergic versus aminergic/excitatory systems in specific behavioral domains. A particularly compelling aspect of this work is the explicit test of predictive validity using compounds already used or seriously considered in human delirium contexts. The authors chose minocycline (an anti-inflammatory and microglia-modulating tetracycline), dexmedetomidine (an α2-agonist sedative widely used in ICUs), and rivastigmine (a cholinesterase inhibitor) [15]. These agents each modulated discrete aspects of the phenotype: behavior, inflammatory cytokines, oxidative stress, microgliosis, and acetylcholine/acetylcholinesterase balance. This is important because predictive validity is often underdeveloped in animal delirium studies, yet it is vital for translational credibility. The NIDUS delirium network guidance explicitly recommends that delirium models be tested against existing or candidate clinical interventions; this manuscript follows that guidance and demonstrates that their ICU-like sepsis model is not merely a ‘stress-plus-sickness’ paradigm but a manipulable system responsive to mechanistically targeted treatments.

## How does this align with broader delirium modeling guidance?

The NIDUS delirium network outlined several priorities for preclinical delirium models that include (1) grounding in clinically relevant risk factors and triggers; (2) multi-domain behavioral assessment of delirium-like features; (3) mechanistic characterization (neuroinflammation, neuronal injury, neurotransmitters); 4) incorporation of translational biomarkers (biofluids, electrophysiology, imaging where feasible); and (5) demonstration of face, construct, and predictive validity. Dominguini et al.’s model comprehensively addresses many of these elements.

At the same time, the study highlights areas for further refinement consistent with these proposed NIDUS recommendations. For example, the current work focuses on young adult male rats; expansion to aged animals, females, and animals with pre-existing cognitive vulnerability (e.g., AD models) would increase construct validity with older, multimorbid ICU populations. Incorporation of more fine-grained attention tasks, quantitative sleep and EEG measures, and longitudinal outcomes beyond 36 h would also help capture the fluctuating course and long-term cognitive consequences that are so characteristic of human delirium as well as aid in identifying desperately needed effective nonpharmacological interventions.

## Conclusion: bringing the ICU to the bench

Dominguini et al. provide compelling evidence that explicitly modeling the ICU environment enhances both the severity and the richness of delirium-like behavioral and neurobiological changes triggered by surgery and sepsis. They conclusively demonstrated that the ICU-like environment worsened behavioral and neurochemical changes, surgery plus anesthesia, and sepsis, supporting its value for enhancing model validity and representing a stepwise improvement in modeling a complex problem. As such, it is a conceptual shift that treats the ICU milieu as a mechanistic contributor to delirium rather than a passive backdrop. By systematically building face, construct, and predictive validity into an ICU-like delirium model, this work directly operationalizes several major recommendations from the contemporary delirium modeling literature and provides a more realistic background to test new interventions. It provides a robust platform on which future studies can layer additional dimensions of vulnerability (age and comorbidities), phenotyping (EEG, sleep, and imaging), and intervention testing. Most importantly, it refocuses attention on the detrimental and potentially modifiable aspects of the ICU environment that directly impact patients. As such, it represents a key step toward more realistic and translationally meaningful models of ICU delirium, opportunities to test pharmacological and nonpharmacological interventions, and brings the ICU to the bench to better serve patients at risk for this devastating complication.

## Abbreviations

CLP: cecal ligation and puncture
dB: decibel
EEG: electroencephalogram
ICU: intensive care unit
IL-6: Interleukin-6
IL-1β: Interleukin 1β
NIDUS: Network for Investigation of Delirium: Unifying Scientists
W: Watts
